# Supplementary material for: Deaza-modification of MR1 ligands modulates recognition by MR1-restricted T cells
Source: Sci Rep. 2022 Dec 29;12:22539. doi: 10.1038/s41598-022-26259-y (PMC9800373; doi:10.1038/s41598-022-26259-y)
Supplement: Supplementary file 1 — Supplementary Figures. [file 41598_2022_26259_MOESM1_ESM.docx]

**Deaza-modification of MR1 ligands modulates recognition by MR1-restricted T cells**

Haihong Jin^1*^, Nicole A. Ladd^2*^, Andrew M. Peev^2^, Gwendolyn M. Swarbrick^3^, Meghan Cansler^3^, Megan Null^3^, Christopher T. Boughter^4^, Curtis McMurtrey^5^, Aaron Nilsen^1,6^, Karen M. Dobos^7^, William H. Hildebrand^8^, Deborah A. Lewinsohn^3,9^, Erin J. Adams^2^, David M. Lewinsohn^3,6,9^, Melanie J. Harriff^6,9,10#^

**Supporting Information**

**Contents:**

**Figure S1:** Comparisons of docking modes in MR1 donor structures

**Figure S2:** Comparison of docked ligands to crystallographically-determined ligand binding mode

**Figure S3:** RMSD of ligands during simulation trajectories

**PDB Files:** Coordinates for donor structures and docked ligands

**Figure S1.** Docking comparisons for relevant donor structure/ligand pairs. a-b) DMRL (red), DZ (blue), 2’-deoxy-DZ (purple) and monomethyl-DZ (yellow) docked into 4GUP (a) and 4L4V (b). c) Comparison of the docking mode for DMRL in 4GUP (red) and 4L4V (maroon). d-e) PLI (mauve) and DZPLI (light pink) docked into 4GUP (d) and 4L4V (e).

**Figure S2.** Comparisons between docking results and experimentally determined structures of ligand-MR1 complexes. The crystal structure ligand is shaded (dark cyan) and the docked ligand structure is vibrant (cyan). a) HMRL docked into WT MR1 (PDB: 4L4V). b) HMRL docked into MR1^K43A^ (PDB: 4LCW). C) 5-OP-RU docked into MR1^K43A^ (PDB: 4NQD). d) rRL-6-CH_2_OH docked into chimeric human-bovine MR1; 4LCC remains coplanar with the experimentally determined structure, but adopts a conformation ~180° flipped relative to that seen in the crystal structure.

**Figure S3.** Root mean square deviation (RMSD) calculations of each ligand within the binding pocket of MR1 highlight their stability within the pocket over the course of each simulation. RMSD for each ligand across the course of each simulation replicate (*i.e.* “DMRL1”) are shown for a) DMRL, b) DZ, c) 2’-deoxy-DZ, and d) monomethyl-DZ.

**PDB Files.** When files are opened in the same session in a software used for viewing molecular coordinates (e.g. PyMOL

or VMD), they will open as aligned for analyses in the main manuscript.

4GUP Donor Structure

**4gup.pdb**: Donor structure of MR1 (originally crystallized with 6-FP)

**4gup_DMRL_dock.pdb**: DMRL docked into the 4GUP donor structure

**4gup_DZ_dock.pdb**: DZ docked into the 4GUP donor structure

**4gup_DDZ_dock.pdb**: 2’-Deoxy-DZ docked into the 4GUP donor structure

**4gup_MMDZ_dock.pdb**: Monomethyl-DZ docked into the 4GUP donor structure

**4gup_PLI_dock.pdb**: PLI docked into the 4GUP donor structure

**4gup_DZPLI_dock.pdb**: DZPLI docked into the 4GUP donor structure

4L4V Donor Structure

**4l4v.pdb**: Donor structure of MR1 (originally crystallized with HMRL and a TCR)

**4l4v_DMRL_dock.pdb**: DMRL docked into the 4L4V donor structure

**4l4v_DZ_dock.pdb**: DZ docked into the 4L4V donor structure

**4l4v_DDZ_dock.pdb**: 2’-Deoxy-DZ docked into the 4L4V donor structure

**4l4v_MMDZ_dock.pdb**: Monomethyl-DZ docked into the 4L4V donor structure

**4l4v_PLI_dock.pdb**: PLI docked into the 4L4V donor structure

**4l4v_DZPLI_dock.pdb**: DZPLI docked into the 4L4V donor structure

Docking Results Comparing Crystallographic Binding

**4l4v.pdb**: Donor structure of MR1 (originally crystallized with HMRL and a TCR; same as above)

**4l4v_HMRL_dock.pdb**: HMRL docked into the 4L4V donor structure

**4l4v_HMRL_xtal.pdb**: crystallographic orientation of HMRL in the 4L4V donor structure

**4nqd.pdb**: Donor structure of MR1^K43A^ (originally crystallized with 5-OP-RU and a TCR)

**4nqd_5OPRU_dock.pdb**: 5-OP-RU docked into the 4NQD donor structure

**4nqd_5OPRU_xtal.pdb**: crystallographic orientation of 5-OP-RU in the 4NQD donor structure

**4lcw.pdb**: Donor structure of MR1^K43A^ (originally crystallized with HMRL and a TCR)

**4lcw_HMRL_dock.pdb**: HMRL docked into the 4LCW donor structure

**4lcw_HMRL_xtal.pdb**: crystallographic orientation of HMRL in the 4LCW donor structure

**4lcc.pdb**: Donor structure of bovine-human MR1 chimera (originally crystallized with heterogeneous assortment of ligands from *E. coli* supernatant and a MAIT TCR)

**4lcc_rrl6CH2OH_dock.pdb**: rRL-6-CH_2_OH docked into the 4LCC donor structure

**4lcc_ rrl6CH2OH_xtal.pdb**: crystallographic orientation of rRL-6-CH_2_OH in the 4LCC donor structure
